# Mass Spectrometry-Based Comparative Evaluation of Forced Glycation Profiles of Therapeutic Monoclonal Antibodies

**DOI:** 10.3390/biomedicines14081683

**Published:** 2026-07-27

**Authors:** Ceren Pamukcu, Ahmet Emin Atik

**Affiliations:** 1 Department of Biomedical Engineering, Faculty of Engineering and Natural Sciences, Acibadem Mehmet Ali Aydinlar University, Atasehir, 34752 Istanbul, Türkiye; cerenpamukcu93@gmail.com; 2 Department of Natural Sciences, Faculty of Engineering and Natural Sciences, Acibadem Mehmet Ali Aydinlar University, Atasehir, 34752 Istanbul, Türkiye; 3 Turgut Ilaclari A.S., 41400 Kocaeli, Türkiye

**Keywords:** forced glycation, biosimilar, originator, glycation profile, mass spectrometry

## Abstract

**Background/Objectives**: Glycation, a non-enzymatic post-translational modification, can increase structural heterogeneity in therapeutic monoclonal antibodies (mAbs) and is considered a critical quality attribute in biosimilar (BS) development. Despite extensive studies on glycation in individual mAbs, comparative reports on originator (OR) and BS mAbs under forced glycation conditions remain limited. This study aimed to comparatively evaluate the glycation profiles of one OR and three BS anti-tumor necrosis factor-alpha (anti TNF-α) mAb products using integrated mass spectrometry-based methods. **Methods**: Forced glycation was induced by incubating mAbs with 200 mM D-glucose at 37 °C for 7 days. Intact mass analysis and peptide mapping were used to assess glycation extent and site distribution, respectively. **Results**: Intact mass analysis revealed a consistent mass increase of approximately 486 Da across all major glycoform species for each mAb product, indicating predominant formation of the tri-glycated mAb population under the applied stress conditions. The overall glycation levels were comparable at the intact level, ranging from 67% to 73% among the OR and BS mAb products. Peptide mapping identified nine glycated lysine (K)-containing peptides, among which three major glycation hotspots (LC:V5 K145/K149, LC:V7 K183, and HC:V7 K250/K252) showed elevated occupancies (~10–16%). These sites collectively accounted for the dominant intact level mass shift. The remaining glycated peptides exhibited only minimal modification levels (<2%). Despite their distinct manufacturing processes, all tested mAb products showed nearly identical site-specific glycation profiles. **Conclusions**: Forced glycation susceptibility in the studied mAbs was driven by a limited set of structurally preferred K hotspots, leading to highly comparable glycation profiles across OR and BS mAb products. The combined intact mass and peptide mapping strategy provides a robust analytical platform for comparative glycation assessment in BS characterization.

## 1. Introduction

Protein glycation is a non-enzymatic and spontaneous post-translational modification (PTM) in which reducing sugars covalently attach mainly to the ε-amino groups of lysine (K) residues and, to a lesser extent, to the N-terminal α-amino groups of proteins [1]. The process begins with a condensation reaction between the carbonyl group of a reducing sugar and a free amino group, yielding an unstable Schiff base (aldimine). This intermediate product then undergoes an Amadori rearrangement to yield a more stable ketoamine product [2]. These reactions are collectively known as the Maillard reaction [2,3]. Glycation changes protein structure, stability, and biological activity, and may lead to the formation of advanced glycation end products (AGEs). These products are associated with protein degradation and various pathological conditions, including diabetic complications [4,5,6]. Exposure of monoclonal antibodies (mAbs) to reducing sugars can also cause glycation at multiple stages of the mAb manufacturing process [7,8,9,10,11,12,13,14]. In the cell culture media of mAbs, glucose serves as the primary carbon and energy source. It is a major contributor to glycation during upstream processing, as secreted mAbs are continuously exposed to it [7,8,9]. Moreover, galactose is often supplemented in cell cultures to modulate mAb fragment crystallizable (Fc) region galactosylation levels [10]. Furthermore, formulation excipients such as sucrose and trehalose may degrade under thermal stress to generate reducing sugars, thereby promoting glycation during storage or handling [11,12,13]. Beyond the manufacturing process, mAbs exhibit low glycation levels under recommended storage conditions (2–8 °C), during administration, and in vivo circulation [13]. Glycation can alter mAbs’ structural integrity and heterogeneity, consequently affecting their biological activity and stability profiles [15,16,17]. Therefore, glycation is considered a critical quality attribute (CQA), particularly when it occurs within complementarity-determining regions (CDRs), because it may influence antigen binding properties [17]. Therefore, a detailed qualitative and quantitative characterization of glycation is important during mAb development processes.

Multiple process parameters, including glucose concentration, feeding strategy, temperature, process duration, and pH, influence the extent and kinetics of mAb glycation during manufacturing [9,16,18,19,20,21]. Yuk et al. [9] demonstrated that controlling glucose concentration and feeding strategies in Chinese hamster ovary (CHO) fed batch cultures minimized glycation, highlighting the importance of upstream process control. Miller et al. [18] showed that solvent accessibility is a key parameter of K susceptibility in which glycation occurs during cell culture and downstream processing. Agarwal et al. [19] also developed a kinetic model that relates glycation rates to parameters such as glucose concentration, temperature, and pH. The authors provided a predictive study for process optimization. Furthermore, Jacobitz et al. [20] demonstrated that formulation buffer composition and pH markedly influenced site-specific glycation rates. Recently, Pappenreiter et al. [21] reported that glycation in CHO-fed batch systems follows a constrained second-order reaction dependent on glucose exposure and culture duration.

Analytical techniques for detecting and quantifying glycation levels include boronate affinity chromatography (BAC), capillary isoelectric focusing (cIEF), ion-exchange chromatography (IEX), and liquid chromatography–mass spectrometry (LC–MS) [7,17,18,22,23,24,25,26,27]. BAC selectively enriches glycated species and is widely used for overall glycation quantification in cell cultures or purified samples [22,23], while cIEF and IEX enable charge-based separation of glycation-induced acidic variants produced during process development and stability studies [17,27]. Nuclear magnetic resonance (NMR) spectroscopy, introduced as an orthogonal technique for glycation analysis, allows direct identification and estimation of glycated K residues [28]. Among these approaches, MS-based methods remain the most powerful, enabling the total and site-specific characterization of glycation. Specifically, glycation increases mass by 162 Da per modification, which intact mass analysis can readily detect, whereas peptide mapping enables high-sensitivity localization and the relative quantification of modified K residues [8,11,16,17,18,24,25,26,29,30].

Because mAb-based therapeutics have high specificity and affinity for target antigens, they have become central to cancer and immune-mediated disease treatment over the past three decades [31]. Patent expirations for several blockbuster originator (OR) products have substantially accelerated biosimilar (BS) development. However, BSs are required to demonstrate a high degree of similarity to their corresponding OR products in terms of quality, efficacy, and safety profiles. OR and BS products share the same primary amino acid sequence but are manufactured using distinct upstream and downstream processes, including different CHO cell culture conditions, glucose feeding strategies, purification steps, and formulation compositions. Such manufacturing-specific process variations drive structural heterogeneity among therapeutic mAbs [32,33] and may potentially influence their susceptibility to PTMs, including glycation. Although glycation in individual mAbs has been increasingly studied, few comparative reports have evaluated whether OR and BS mAb products exhibit similar glycation behaviors under accelerated stress conditions. In particular, whether process-related differences affect the extent and site-specific distribution of glycation under forced conditions remains insufficiently understood. Therefore, in this study, one OR and three BS mAb products were comparatively evaluated under forced glycation conditions using integrated LC–MS approaches. Two of the BSs were commercially available products from different manufacturers, and the third was a BS candidate developed by Turgut Ilaclari A.S.

## 2. Materials and Methods

### 2.1. Materials and Reagents

The OR and three BS products (BS1, BS2, and BS3) are humanized anti-tumor necrosis factor-alpha (anti TNF-α) IgG mAbs. The OR, BS1, and BS2 were commercially sourced from their respective manufacturers, whereas BS3 was developed as a BS candidate by Turgut Ilaclari A.S. (Istanbul, Türkiye). All mAb products were stored at 4 °C until use.

All reagents used were of analytical grade or higher. Acetonitrile, trifluoroacetic acid (TFA), formic acid (FA), and D-glucose were purchased from Merck (Darmstadt, Germany). Ammonium bicarbonate, guanidine hydrochloride, dithiothreitol (DTT), iodoacetamide (IAM), sodium iodide, and histidine monohydrochloride monohydrate were obtained from Sigma-Aldrich (St. Louis, MO, USA). Leucine enkephalin was purchased from Waters (Milford, MA, USA), and sequencing-grade endoproteinase Glu-C (from *Staphylococcus aureus* V8) was purchased from Promega (Madison, WI, USA). Ultrapure water (18.2 MΩ·cm) was prepared in-house using a Milli-Q Direct 8 purification system (Merck Millipore, Darmstadt, Germany). A 50 kDa molecular weight cutoff (MWCO) centrifugal filter unit (Amicon^®^ Ultra-0.5) was obtained from Merck Millipore (Burlington, MA, USA).

### 2.2. Preparation of Forced-Glycated mAb Samples

All mAb products were buffer exchanged into 100 mM ammonium bicarbonate and adjusted to a final concentration of 10 mg/mL. This step was performed to standardize the reaction conditions across mAb products by minimizing formulation-dependent effects. Forced glycation was performed by incubating equal volumes of mAb solution with 200 mM D-glucose (prepared in ultrapure water) at 37 °C for 7 days in the dark. Control samples were incubated under identical conditions in the absence of glucose. After 7 days of incubation, samples were buffer exchanged using 50 kDa MWCO centrifugal filter units to remove excess glucose and stored at ≤−65 °C until analysis.

### 2.3. Sample Preparation for Intact Mass Analysis

The control and glycated mAb samples were diluted to 1 mg/mL with 50 mM ammonium bicarbonate solution. A 2 μL aliquot of each sample was individually injected onto a C4 column (Waters Acquity UPLC Protein BEH C4, 300 Å, 1.7 μm, 2.1 × 50 mm) for chromatographic separation. Mobile phase A comprised 0.1% FA in water, and mobile phase B comprised 0.1% FA in acetonitrile. Separation was achieved using a 5-min gradient, increasing mobile phase B from 5% to 90% over 2.7 min, at a flow rate of 0.2–0.5 mL/min. The column temperature was maintained at 80 °C, and eluting species were monitored by UV absorbance at 280 nm prior to MS detection. The reported values are the mean ± standard deviation (SD) of the three preparations.

### 2.4. Sample Preparation for Peptide Mapping Analysis

Each mAb sample was diluted to 1 mg/mL with ultrapure water, and a 10 μL aliquot was denatured with 8 M guanidine hydrochloride. Reduction was performed with 500 mM DTT for 30 min at 57 °C, followed by alkylation with 300 mM IAM at room temperature for 30 min in the dark. The reduced and alkylated samples were buffer exchanged into 200 mM histidine monohydrochloride monohydrate buffer (pH 6.0) using a desalting column. Enzymatic digestion was conducted using a sequencing-grade Glu-C protease at an enzyme-to-protein ratio of 1:50 (*w*/*w*) at 37 °C for 18 h. After overnight digestion, the reaction was quenched by acidification with 50% TFA, and the insoluble material was removed by centrifugation. A 45 μL aliquot of the supernatant was injected onto a C18 column (Waters Acquity UPLC Peptide BEH C18, 300 Å, 1.7 μm, 2.1 × 100 mm) for the chromatographic separation of peptides by gradient elution. The column temperature was held at 40 °C during the analysis. Peptides were separated over a 90-min gradient, including a 70 min linear gradient from 5% to 60% acetonitrile at a flow rate of 0.25 mL/min. Mobile phase A comprised 0.1% TFA in water, and mobile phase B comprised 0.1% TFA in acetonitrile. Although TFA is known to reduce ESI efficiency, it was selected because it provides better separation of the peptides. Eluted peptides were monitored at 214 nm and analyzed using online coupled high-resolution tandem mass spectrometry (MS/MS). Samples were prepared in triplicate, and the reported values represent the mean ± SD of three preparations.

### 2.5. Mass Spectrometry

LC–MS and LC–MS/MS analyses were performed using an Acquity UPLC^®^ H-Class Bio system coupled online to a Xevo G2–XS QToF hybrid mass spectrometer (Waters, Milford, MA, USA). Data were acquired in positive electrospray ionization (ESI) mode. For intact mass analysis, MS scans were acquired over an *m*/*z* range of 400–4000. For peptide mapping, MS^E^ data were acquired over *m*/*z* 50–2000. For intact mass analysis, the capillary and cone voltages were 3 kV and 30 V, with source and desolvation temperatures of 150 °C and 500 °C, respectively. For peptide mapping, the capillary and cone voltages were set to 1 kV and 25 V, with source/desolvation temperatures of 100 °C and 350 °C. Sampling cone and desolvation gas flow rates were set to 50 L/h and 800 L/h for both analyses, and the autosampler temperature was maintained at 10 °C. External calibration was performed prior to analysis using sodium iodide solution (2 μg/μL). Leucine enkephalin was used as a lock mass for real-time mass correction. Instrument control, data acquisition, and processing were performed using UNIFI™ software (v1.9.4, Waters, Milford, MA, USA). Deconvolution of the intact mass spectra was performed using the Maximum Entropy (MaxEnt1™, Waters, Milford, MA, USA) algorithm with 20 iterations. The start and end peak widths were set to 2.4 and 3.5, respectively, and the minimum intensity ratio was set to 80%. The deconvoluted mass range was set to 147,000–151,000 Da. Peptides were identified by matching experimental masses to theoretical values generated in silico from the target protein sequence. MS^E^ acquisition enabled simultaneous collection of precursor and fragment ion data using low (4 V) and elevated (25–40 V) collision energies. PTM localization was based on precursor mass accuracy (±8 ppm) and at least two sequence-specific fragment ions. Carbamidomethylation of cysteine residues was set as a fixed modification, and K glycation was defined as a variable modification during data processing.

### 2.6. Statistical Analysis

Statistical analyses were performed using GraphPad Prism v9.1.3 (San Diego, CA, USA). A non-parametric Kruskal–Wallis test with 95% confidence was performed to compare glycation levels. Multiple comparisons were tested with Dunn’s statistical hypothesis testing. Data are presented as the mean value ± SD, and statistical significance was defined as *p* < 0.05.

## 3. Results

### 3.1. Determination of Total Glycation Level by LC–MS Intact Mass Analysis

**Figure 1:**
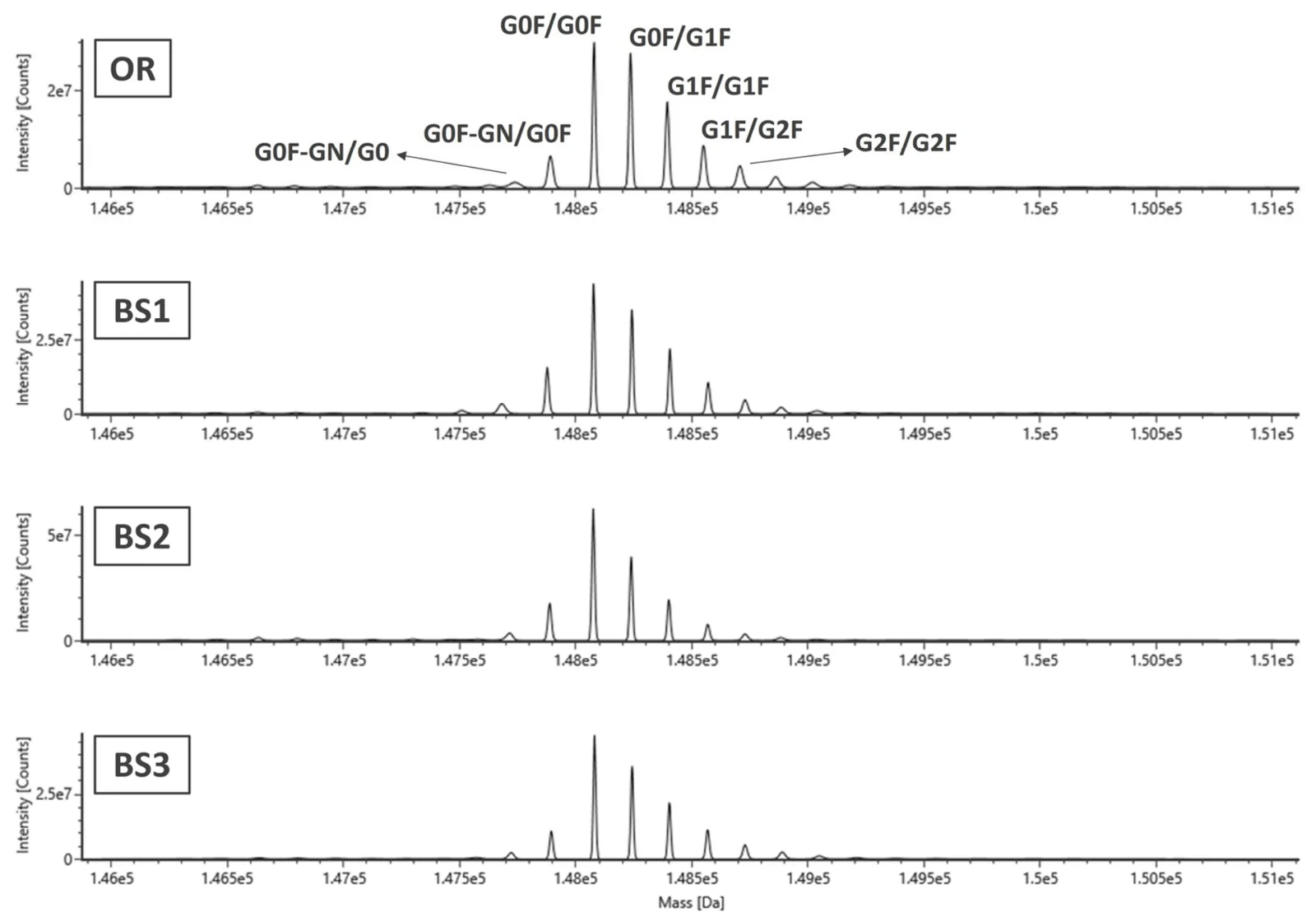
Deconvoluted intact mass spectra of the control OR and BS mAb products.

The first part of the study involved evaluating glycation extent in OR and BS mAb products using intact mass LC–MS analysis. This approach is typically used to determine the total molecular mass of antibodies, including their glycoform distributions. For this purpose, non-glycated (control) and forced-glycated samples were prepared. To have native glycoform profiles, intact mass spectra of the control mAb samples were first acquired. The overlaid raw spectra (Figure S1 in the Supplementary File) within the *m*/*z* range of 2000–4000 exhibited symmetrical charge state distributions centered at *z* = 52–54 for all products. Zoomed-in views of the most intense region (Figure S2 in the Supplementary File) confirmed highly similar glycoform patterns across the OR and BS products. Deconvolution of the raw spectra using the full charge state distribution generated zero-charge mass spectra (see Figure 1), enabling direct comparison of glycoform masses.

As expected, Fc *N*-linked glycosylation primarily contributed to molecular heterogeneity in all tested samples. Seven major glycoforms, including G0F-GN/G0, G0F-GN/G0F, G0F/G0F, G0F/G1F, G1F/G1F (or G0F/G2F), G1F/G2F, and G2F/G2F, were identified in the OR and BS products. The intact mass spectra demonstrated a high degree of similarity across all samples (Figure 1), with comparable glycoform distributions and relative abundances. As listed in Table 1, the observed masses closely matched the expected values (A maximum mass error tolerance of 200 ppm was applied for glycoform assignment during intact mass analysis).

**Figure 2:**
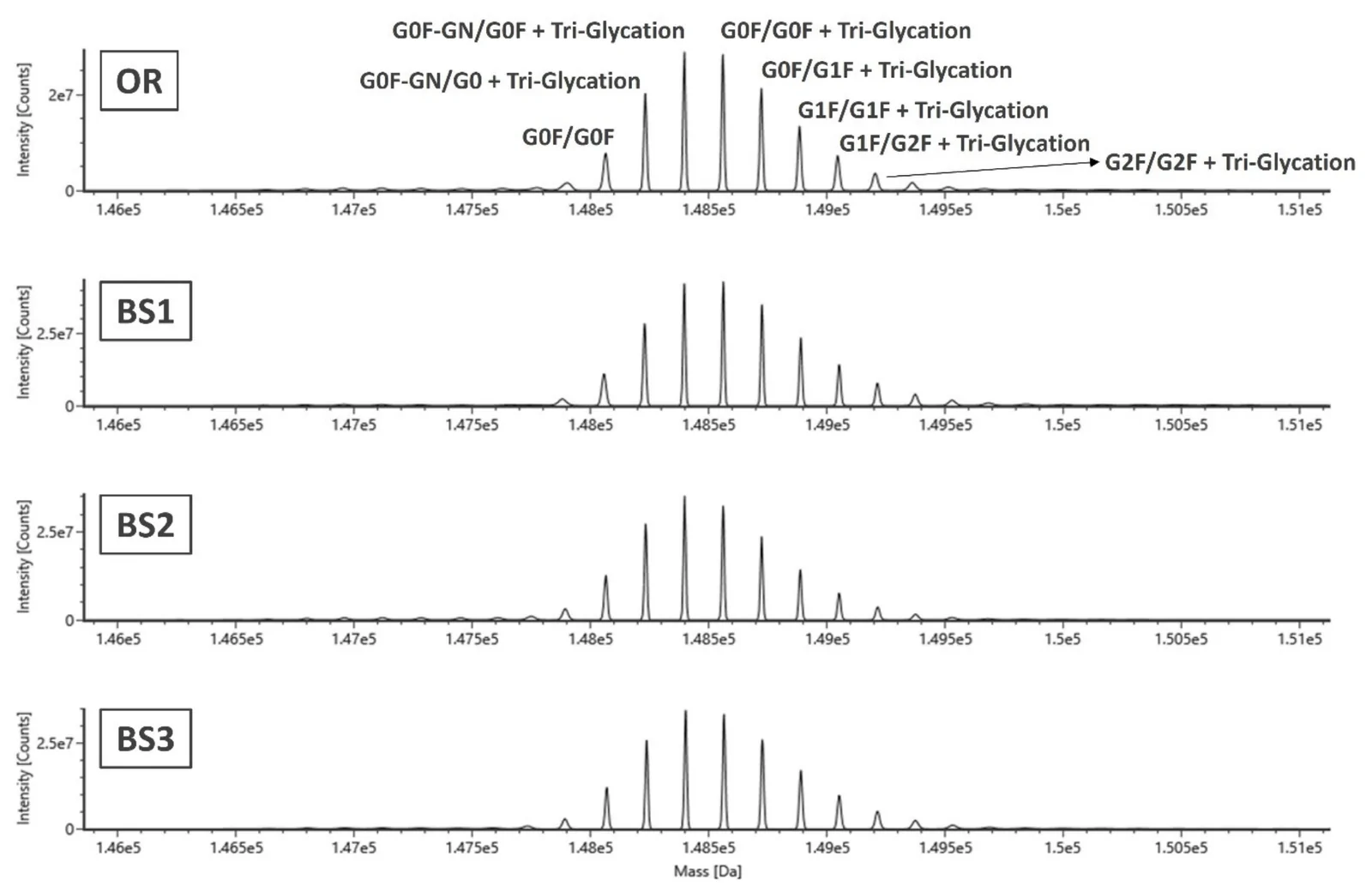
Deconvoluted intact mass spectra of the forced-glycated OR and BS mAb products.

Following incubation with glucose solution at 37 °C for 7 days, intact mass spectra of glycated samples were acquired and compared with their control counterparts. Total ion chromatograms (TICs) indicated that control and glycated samples exhibited identical retention times (~2.6 min), suggesting no significant change in chromatographic behavior upon glycation. As given in the Supplementary File, the overlaid raw spectra of glycated samples (Figure S3) and corresponding zoomed regions (Figure S4) revealed a shift in the charge state distribution of the dominant peaks. Specifically, the charge state envelopes of glycated species were shifted by approximately one charge unit toward lower *m*/*z* values (i.e., higher charge states), consistent with an increase in molecular mass due to covalent glucose addition (+162 Da per modification). Deconvolution of these spectra yielded zero-charge mass profiles for each sample (see Figure 2). Comparison of the control and glycated intact mass spectra demonstrated a consistent mass increase for all major glycoform species following forced glycation. For example, the G0F/G0F glycoform increased from 148,078.04 Da (control) to 148,559.99 Da (glycated), corresponding to an experimental mass increase of +482 Da for the OR product (Table 1 and Table 2). Since the theoretical mass increment associated with one glucose residue is approximately 162 Da, the experimentally observed mass shift is in close agreement with the expected increase of +486 Da for three glucose additions (tri-glycation). The minor deviation (4 Da) likely resulted from deconvolution uncertainty and instrumental mass accuracy limitations. Similar mass increases were observed for all remaining glycoforms for OR product namely; G0F-GN/G0 (147,736.75 Da to 148,231.45 Da), G0F-GN/G0F (147,890.35 Da to 148,396.87 Da), G0F/G1F (148,235.23 Da to 148,722.50 Da), G1F/G1F (148,393.20 Da to 148,884.60 Da), G1F/G2F (148,549.63 Da to 149,045.94 Da), and G2F/G2F (148,706.07 Da to 149,204.84 Da). Notably, all BS products exhibited comparable mass increase across the major glycoform distributions, with tri-glycated species predominating under the applied forced glycation conditions. It should be emphasized that due to the isobaric nature of glucose addition and terminal galactose residues, alternative glycoform/glycation combinations cannot be completely excluded. Overall, the intact mass profiles of BS1, BS2, and BS3 closely matched those of the OR product, demonstrating a highly comparable glycation profile at the intact protein level.

Despite extensive glycation, a minor fraction of non-glycated G0F/G0F species remained detectable in all samples (see Figure 2). The extent of glycation at the intact protein level was calculated semi-quantitatively by comparing the relative abundance of the G0F/G0F glycoform in the control with that of the corresponding G0F/G0F glycoform after forced glycation. This approach provides a relative estimate based on deconvoluted intact mass spectra. The results indicated non-glycation levels of 32.2 ± 0.8% for the OR product, and 33.1 ± 0.6%, 28.2 ± 0.7%, and 27.3 ± 0.8% for BS1, BS2, and BS3, respectively, after incubation at 37 °C for 7 days. The intact non-glycated mAb population exhibited a statistically significant difference among the compared samples (*p* = 0.0015), indicating an altered distribution of unmodified antibody species at the whole-molecule level. Overall, the intact mass data consistently support a predominant antibody population exhibiting a mass increase corresponding to approximately three glucose additions. Because intact mass analysis solely provides information on the total molecular mass change, site-specific peptide mapping was subsequently conducted to identify the susceptible glycation sites. 

### 3.2. Identification of Glycation Sites by LC–MS/MS Peptide Mapping Analysis

The second part of the study used LC–MS/MS peptide mapping to confirm the glycation origin of the mass shift observed in the deconvoluted spectra of the mAb products and to identify and quantify the modified K residue site specifically. For this purpose, 7-day forced glycation samples were denatured, reduced, alkylated, and subsequently subjected to enzymatic digestion. Despite its widespread use in peptide mapping workflows, trypsin is unsuitable for glycation analysis. The glycation on the K residue inhibits proteolytic cleavage, leading to missed cleavages. Therefore, endoproteinase Glu-C was employed to enable reliable identification and quantification of glycation sites. Glu-C specifically cleaves at the C-terminal side of glutamic acid (E) residues. The model mAb contains 13 K residues in the light chain (LC) and 30 in the heavy chain (HC) (excluding the C-terminal K variant), all of which are potential glycation sites. However, glycation is site-selective and depends on factors such as solvent accessibility, steric constraints, and the local chemical environment [11,16,20]. Table 3 lists nine glycated peptides identified under forced glycation conditions in the OR and BS mAb products. Glycation levels were quantified by integrating extracted ion chromatogram (XIC) peak areas and expressed as the percentage of glycated peptide relative to the total (glycated + non-glycated) peptide signal. This calculation should be regarded as a semi-quantitative estimation because it was derived from XIC peak areas without correction for potential differences in ionization efficiency between glycated and non-glycated peptides. Although four peptides contained multiple K residues, only a single glycation event per peptide was observed, indicating site-specific mono-glycation. Notably, no glycation was detected at the N-termini of either LC or HC. All identified glycated peptides were within ±4.0 ppm mass error.

Six peptides (LC:V3, LC:V4, LC:V8, HC:V10, HC:V15, and HC:V20) exhibited low glycation levels (<2%) across all products (Table 3). Their identification was primarily based on accurate mass measurements, as shown in the Supplementary File (Table S1). Low signal intensity limited MS/MS confirmation of these peptides. Owing to their low abundance, these peptides are not expected to contribute markedly to intact mass profiles. In contrast, three peptides, namely LC:V5, LC:V7, and HC:V7, showed substantially higher glycation levels (~10–16%) and were therefore considered the major contributors to the glycation pattern observed at the intact protein level. Accurate mass measurements (see Table 4) and MS/MS fragmentation confirmed the amino acid sequences of these three peptides. The relatively poor MS/MS spectra obtained for glycated peptides in our study are attributed mainly to the low abundance of the modified species and the charge state distribution of ions selected for collision-induced dissociation (CID) analysis. Moreover, under reversed-phase LC conditions, glycated peptides eluted slightly earlier (by ~0.2–0.5 min) than their unmodified counterparts. The added sugar moiety increases peptide hydrophilicity, which can explain the observed retention shift.

Importantly, the combined occupancies of the three major glycated peptide regions (LC:V5, LC:V7, and HC:V7) closely matched the tri-glycation pattern observed at the intact protein level. This correlation suggests that the predominant +486 Da mass increase mainly arises from glycation at a few preferred regions rather than from low-level modifications throughout the antibody sequence. These findings further indicate that forced glycation preferentially targets structurally favored hotspots.

**Figure 3:**
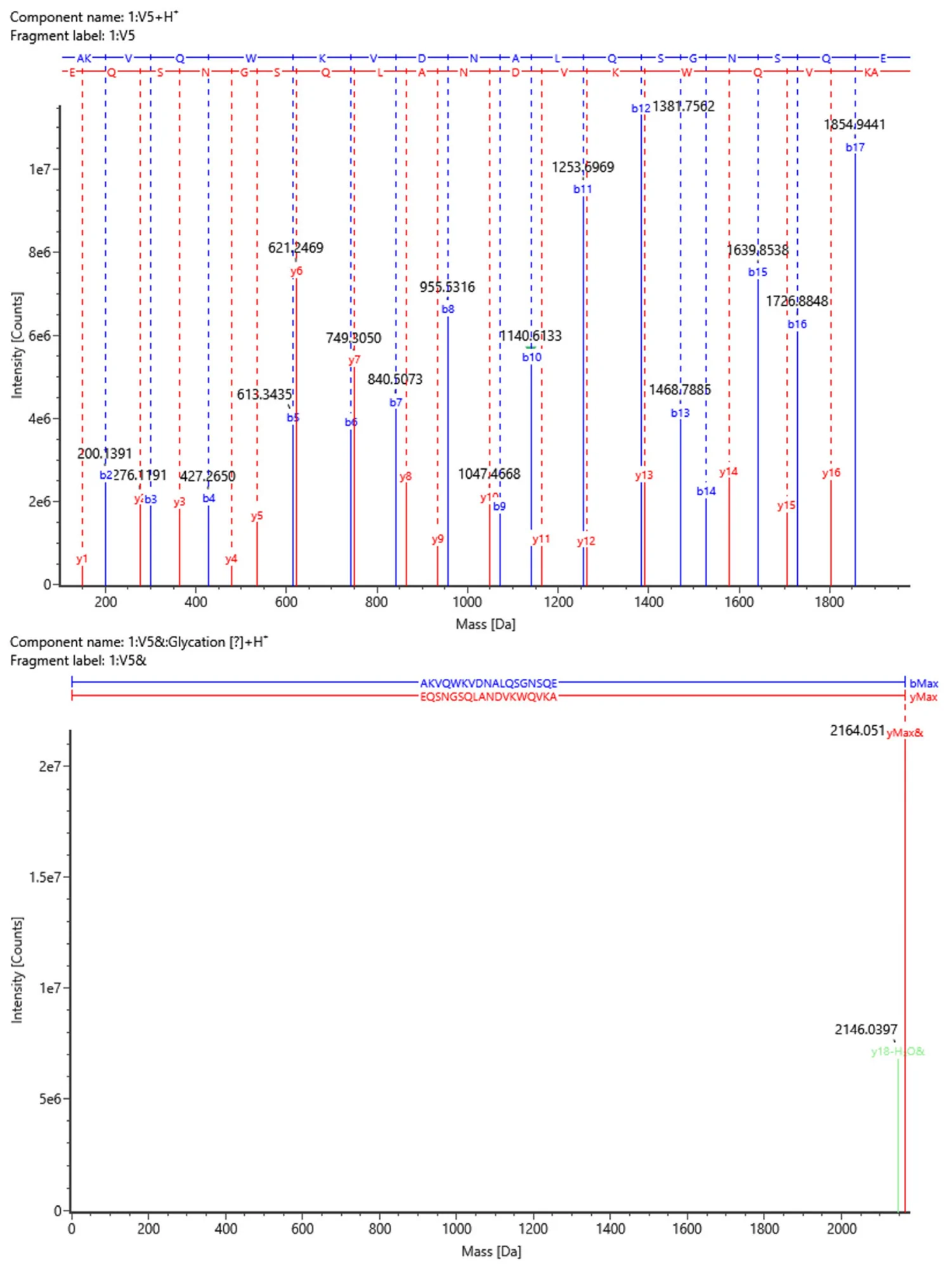
Representative MS/MS spectra of the LC:V5 peptide obtained from the control ((**upper**) panel) and forced-glycated ((**lower**) panel) mAb products.

For the LC:V5 peptide (AKVQWKVDNALQSGNSQE; expected mass: 2001.9989 Da), the unmodified form in the OR sample was detected at 2001.9983 Da (−0.2 ppm). Mono-glycation at either K145 or K149 resulted in an observed mass of 2164.0525 Da (expected: 2164.0517 Da; 0.4 ppm), consistent with the addition of one hexose (+162.0528 Da). As shown in Figure 3, MS/MS analysis of the unmodified peptide showed extensive *b*- and *y*-ion series, whereas the glycated peptide produced only two major fragment ions (*y*_18_ and *y*_18_–H_2_O), confirming mono-glycation but not allowing site localization between K145 and K149. Table 4 compares the expected and observed masses of the BS samples and their corresponding mass errors. Glycation levels for LC:V5 were 9.71 ± 0.69% (OR) and 10.0 ± 0.78%, 10.2 ± 0.63%, and 9.46 ± 0.58% for BS1, BS2, and BS3, respectively (Table 3).

**Figure 4:**
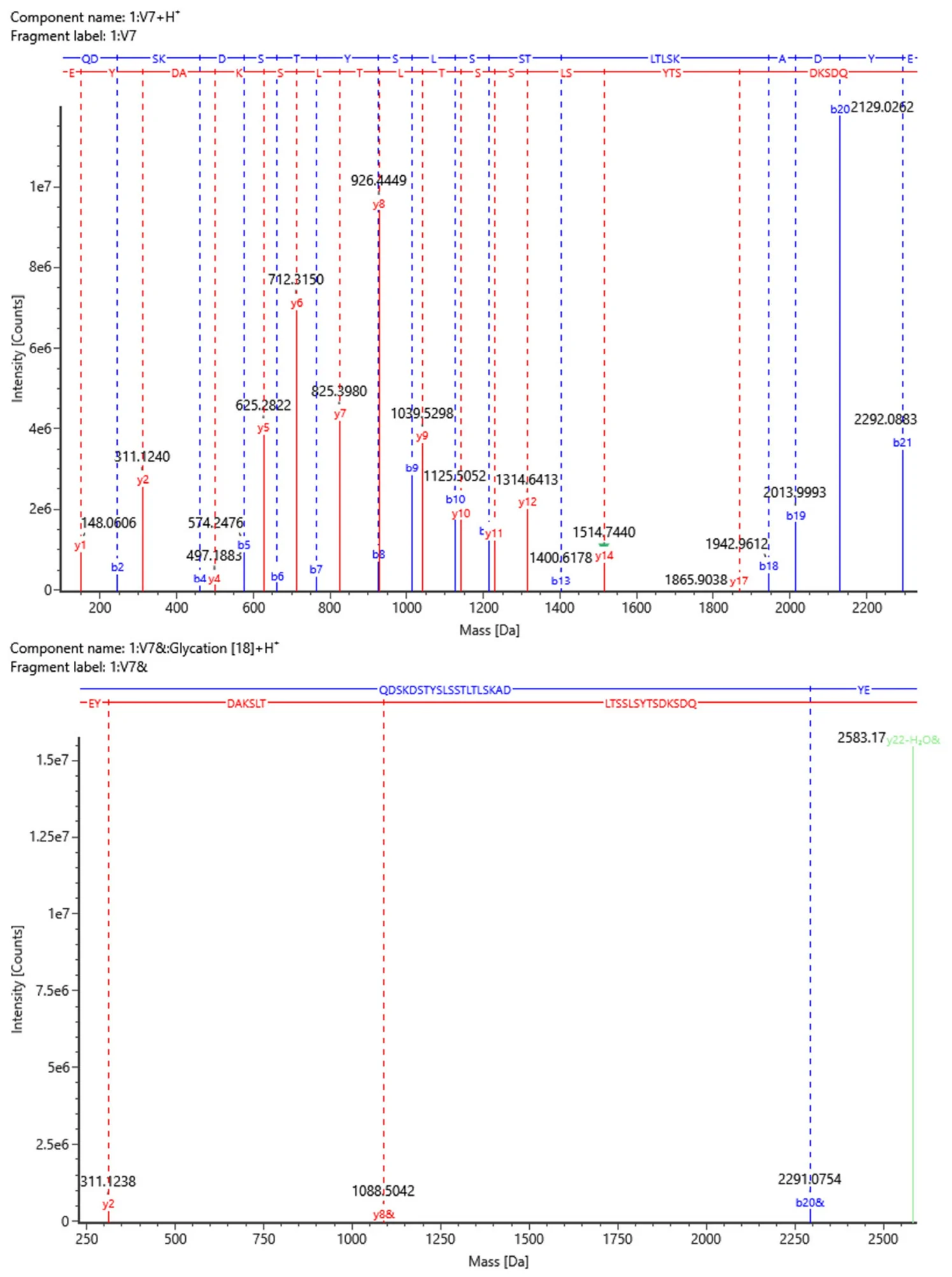
Representative MS/MS spectra of the LC:V7 peptide obtained from the control ((**upper**) panel) and forced-glycated ((**lower**) panel) mAb products.

The most extensive glycation level was observed for the LC:V7 peptide (QDSKDSTYSLSSTLTLSKADYE; expected mass of unmodified peptide: 2439.1409 Da). In the OR sample, the glycated form was detected at 2601.1938 Da (0 ppm), corresponding to mono-glycation. Among the two K residues present (K169 and K183) in the sequence, MS/MS analysis confirmed that glycation occurs at the K183 residue (Figure 4). Comparative fragment ion analysis showed approximately 162.05 Da shifts for *y*_8_ (1088.5042 Da) and *b*_20_ (2291.0754 Da) ions relative to their unmodified counterparts (*y*_8_, 926.4449 Da; *b*_20_, 2129.0262 Da), confirming glucose-mediated modification at this site. Table 4 compares the expected and observed masses and their corresponding mass errors. The calculated glycation levels at K183 were 16.0 ± 1.00% for OR and 16.6 ± 1.01%, 15.4 ± 0.87%, and 15.7 ± 0.92% for BS1, BS2, and BS3, respectively (see Table 3).

**Figure 5:**
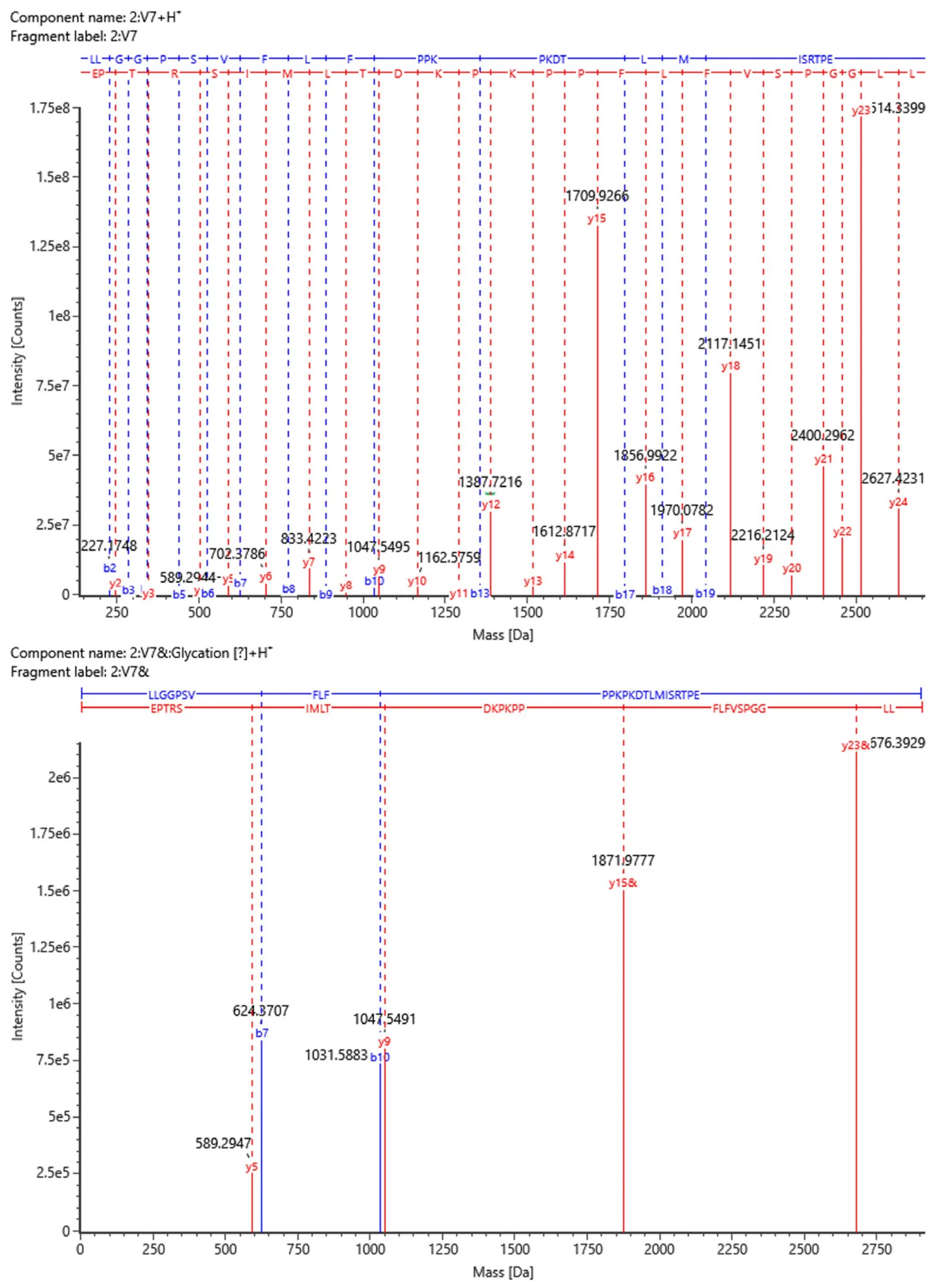
Representative MS/MS spectra of the HC:V7 peptide obtained from the control ((**upper**) panel) and forced-glycated ((**lower**) panel) mAb products.

The HC:V7 peptide (LLGGPSVFLFPPKPKDTLMISRTPE; expected mass: 2740.5106 Da) represented the second most glycated site (~14%). The mono-glycated form was observed at 2902.5640 Da (0.2 ppm) for OR. Although the peptide contains two K residues, K250 and K252, low-quality MS/MS data under CID conditions hindered exact glycation-site assignment. Nevertheless, the presence of diagnostic fragment ions, namely *y*_15_ (1871.9777 Da) and *y*_23_ (2676.3929 Da), confirmed the glycation of this peptide (Figure 5). As listed in Table 3, the glycation levels were 13.7 ± 0.92% (OR) and 14.2 ± 0.71%, 14.0 ± 0.80%, and 13.3 ± 0.87% for BS1, BS2, and BS3, respectively.

Statistical evaluation of the relative abundances of the glycation hotspots on LC:V5, LC:V7, and HC:V7 peptides demonstrated no significant differences among the compared samples, with *p*-values of 0.55, 0.47, and 0.61, respectively (*p* > 0.05). Overall, peptide mapping demonstrated highly comparable K glycation extent and site distribution between the OR and BS products. Glycation was observed at varying levels across specific peptides following forced glycation, whereas no glycated residues were detected in control (non-stressed) samples.

## 4. Discussion

Glycation is a non-enzymatic PTM that can affect the structural integrity, biological activity, and pharmacokinetic properties of therapeutic mAbs. For this reason, it is generally accepted as a CQA in BS development and requires careful monitoring to ensure comparability, product quality, and manufacturing consistency [7,23,24]. Beyond the manufacturing steps, glycation may also occur during storage of mAbs in formulation buffers and through administration, as antibodies circulate in vivo [14,34]. However, glycation levels under these conditions are typically low. Therefore, mAbs are commonly subjected to forced glycation (incubation with high concentrations of glucose) to accelerate the reaction and identify glycation-prone sites. Such studies not only enable the identification of susceptible regions and the assessment of glycation extent but also support the development of robust control strategies and comprehensive comparability assessments between OR and BS products under stressed conditions.

In the present study, one OR and three BS mAbs were comparatively evaluated under forced glycation conditions using integrated LC–MS workflows. BS1 and BS2 have regulatory approval from the U.S. Food and Drug Administration (FDA) and the European Medicines Agency (EMA), whereas BS3 is a BS candidate without regulatory approval yet. Consistent with previous studies, intact mass analysis and peptide mapping were employed for qualitative and quantitative glycation assessment [8,17,18,35,36]. To the best of our knowledge, direct comparative studies evaluating forced glycation at both intact protein and peptide levels across an OR and multiple marketed and developmental BS mAbs remain limited. By combining these orthogonal analytical approaches, the present study not only confirms previously recognized glycation-prone regions but also demonstrates that forced glycation produces highly comparable glycation profiles among structurally similar products despite their distinct manufacturing and formulation backgrounds. In principle, differences in upstream glucose exposure, cell culture duration, purification process, and excipient composition may generate subtle variations in higher-order structure and microheterogeneity among products. However, the highly similar glycation profiles observed in this study suggest that inherent antibody structural properties, rather than manufacturing-related differences, mainly influence glycation susceptibility. The glycation behavior of K residues is likely defined by its solvent accessibility, local conformational flexibility, and microenvironmental nucleophilicity.

The deconvoluted intact mass spectrum of the OR product (Figure 2) revealed the addition of approximately three glucose units (~486 Da) to each glycoform species after 7 days of incubation at 37 °C. For the OR product, this was evidenced by the mass increase in the G0F/G0F glycoform from 148,078.04 Da to 148,559.99 Da (Table 1 and Table 2). The nearly identical (~486 Da) mass increase observed across all major glycoforms provides strong evidence for the addition of three glucose residues per antibody under the applied forced glycation conditions. Comparable tri-glycated glycoforms were also detected for BS1, BS2, and BS3. As shown in Figure 1 and Figure 2, all products exhibited similar forced glycation profiles, despite differences in their manufacturing processes.

Notably, enzymatic deglycosylation was not performed; therefore, the theoretical isobaric overlap between glucose addition and terminal galactose heterogeneity, each contributing a +162 Da mass increment, could not be fully eliminated at the intact mass level. Therefore, intact mass assignment of tri-glycation has a certain level of uncertainty when evaluated alone. For example, the addition of three glucose units to the predominant G0F/G0F glycoform (+486 Da) theoretically overlaps with the mass of a G1F/G2F glycoform. Although enzymatic deglycosylation (e.g., PNGase F) or subunit-based approaches (IdeS digestion) are generally employed to eliminate this ambiguity [8,37], these strategies were beyond the scope of the present study. This study was designed to evaluate directly at the intact antibody level while preserving the native glycoform distribution. Nevertheless, glycation assignments and the comparative conclusions of this study are supported by two strong pieces of evidence: (i) the native glycoform distributions remained unchanged following forced glycation treatment (Figure 1 and Figure 2), (ii) a consistent +486 Da mass shift corresponding to tri-glycation was detected across all glycoforms.

Even after 7 days of incubation, a fraction of non-glycated G0F/G0F species remained detectable in all mAb products (Figure 2). Overall, non-glycation levels ranged from 27% to 33%, indicating that a comparable proportion of each mAb product remained non-glycated under the applied conditions. These findings corroborate previous reports [18]. In addition, comparison of raw mass spectra for control and glycated samples (Figures S2 and S4 in the Supplementary File) revealed peak broadening in glycated species, which results from the attachment of glucose units to the mAb structure.

To further characterize site-specific glycation, LC–MS/MS peptide mapping was performed. Glycation at K residues hindered tryptic cleavage, leading to missed cleavages; therefore, endoproteinase Glu-C was employed. Peptide mapping identified nine glycation-prone peptides containing K residues—five in the LC and four in the HC. The extent of glycation varied among sites and correlated with solvent accessibility. The surface-exposed K residues were more susceptible, whereas structurally buried residues were largely protected. This observation is consistent with previous studies demonstrating that glycation predominantly occurs at solvent-accessible K residues [11,38]. Among the identified sites, six peptides (LC:V3, LC:V4, LC:V8, HC:V10, HC:V15, and HC:V20) exhibited low glycation levels (<2%, see Table 3). This amount of site occupancy is unlikely to produce a clearly resolved +162 Da peak in typical intact mass deconvoluted spectra. In contrast, three peptides (LC:V5, LC:V7, and HC:V7) showed higher modification levels (~10–16%). Their sequences were confirmed by matching observed precursor masses with theoretical values and by the analysis of sequence-specific *b*- and *y*-type fragment ions (Figure 3, Figure 4 and Figure 5). The combined contribution of these highly modified sites likely dominates the intact mass profile, resulting in the observed +486 Da shift corresponding to the addition of three glucose units. Specifically, K183 in LC:V7 was identified as the most susceptible glycation site (~16%). Moderate glycation levels were observed at K145/K149 in LC:V5 (~10%) and K250/K252 in HC:V7 (~14%), consistent with a previous report by Quan et al. [7], where the authors determined the same K positions on their tested mAb. The model antibody used in our study contains numerous theoretically modifiable K residues; peptide mapping demonstrated that only three peptide regions display extensive glycation. Intact mass and peptide mapping assays demonstrated that forced glycation did not proceed through uniform modification of all accessible K residues but rather through preferential glucose addition at a few structurally favored sites. Such behavior corroborates previous reports emphasizing the critical role of solvent exposure and local residue environment in determining K reactivity [11,16,20]. Moreover, particular attention was given to determining K residues within CDRs; however, none of the identified glycation sites were located within these regions.

Statistical evaluation showed that the glycation levels of the LC:V5, LC:V7, and HC:V7 peptides did not differ significantly among the mAb products compared. However, a significant difference was observed in the relative abundance of the intact non-glycated mAb population. This finding suggests that while the major glycation-susceptible sites display similar modification behavior across samples, the accumulation of multiple minor glycation events throughout the antibody structure affects the overall level of unmodified intact molecules. It should be noted that intact mass analysis and peptide mapping provide complementary information and therefore should not be expected to yield identical quantitative estimates of glycation. Intact mass analysis reports the distribution of intact antibody according to the total number of incorporated glucose residues, whereas peptide mapping determines the occupancy of individual glycated peptide regions.

## 5. Conclusions

The present study demonstrates that an integrated LC–MS strategy combining intact mass analysis and peptide mapping enables a robust comparative assessment of forced glycation profiles in therapeutic mAbs. Under glucose stress, all evaluated anti-TNFα mAb products exhibited highly similar glycation patterns despite their distinct manufacturing origins, indicating that glycation susceptibility is primarily associated with intrinsic conformational features rather than process-dependent variations. The predominant tri-glycation pattern observed at the intact protein level was consistent with preferential glycation of a limited number of major glycation-prone peptide regions identified by peptide mapping, whereas the remaining susceptible peptides displayed only minor occupancies. Moreover, the absence of detectable glycation within the CDR regions suggests that glucose-mediated modification preferentially targets structurally exposed non-CDR K residues. Collectively, these findings demonstrate comparable forced glycation susceptibility between the OR and BS products and highlight the complementary value of intact mass analysis and peptide mapping for comparative characterization of therapeutic mAbs. These analyses provide structural information on the glycation susceptibility; however, they do not directly assess the biological consequences of these modifications. A detailed evaluation of glycation should integrate structural characterization with orthogonal functional assays to establish the relationship between glycation and biological activity.

## Figures and Tables

**Table 1:** Comparison of the expected glycoform masses with the experimentally observed masses in the control OR and BS mAb products.

Glycoform Type	Expected Mass (Da)	Observed Mass (Da)			
		OR	BS1	BS2	BS3
G0F-GN/G0	147,730.47	147,736.75	147,695.12	147,713.19	147,720.76
G0F-GN/G0F	147,876.61	147,890.35	147,876.29	147,886.93	147,893.88
G0F/G0F	148,079.81	148,078.04	148,075.90	148,074.64	148,080.35
G0F/G1F	148,241.95	148,235.23	148,241.11	148,237.90	148,241.85
G1F/G1F	148,404.09	148,393.20	148,404.90	148,400.28	148,402.35
G1F/G2F	148,566.23	148,549.63	148,569.34	148,567.57	148,567.09
G2F/G2F	148,728.37	148,706.07	148,728.78	148,728.62	148,728.43

**Table 2:** Comparison of the expected glycoform masses with the experimentally observed masses in the forced-glycated OR and BS mAb products.

Glycoform Type	Expected Mass (Da)	Observed Mass (Da)			
		OR	BS1	BS2	BS3
G0F/G0F (No Glycation)	148,079.81	148,063.36	148,060.25	148,064.42	148,068.57
G0F-GN/G0 + Tri-Glycation	148,216.89	148,231.45	148,228.53	148,233.21	148,237.52
G0F-GN/G0F + Tri-Glycation	148,363.04	148,396.87	148,396.41	148,398.27	148,399.75
G0F/G0F + Tri-Glycation	148,566.23	148,559.99	148,561.60	148,561.08	148,564.98
G0F/G1F + Tri-Glycation	148,728.37	148,722.50	148,725.79	148,724.01	148,727.21
G1F/G1F + Tri-Glycation	148,890.51	148,884.60	148,889.60	148,887.79	148,890.02
G1F/G2F + Tri-Glycation	149,052.65	149,045.94	149,052.57	149,052.31	149,052.70
G2F/G2F + Tri-Glycation	149,214.79	149,204.84	149,214.55	149,215.71	149,214.81

**Table 3:** Comparison of the glycation levels of identified glycated peptides in the OR and BS mAb products. Data are presented as mean ± SD (*n* = 3). The glycation site within each peptide sequence is highlighted in red.

Chain:Peptide No	Sequence	Glycation Position	% Glycation (Mean ± SD)			
			OR	BS1	BS2	BS3
LC:V3	IKRTVAAPSVFIFPPSDE	K107	0.16 ± 0.07	0.14 ± 0.05	0.10 ± 0.04	0.15 ± 0.04
LC:V4	QLKSGTASVVCLLNNFYPRE	K126	1.09 ± 0.12	0.86 ± 0.12	0.71 ± 0.20	0.96 ± 0.14
LC:V5	AKVQWKVDNALQSGNSQE	K145/K149	9.71 ± 0.69	10.0 ± 0.78	10.2 ± 0.63	9.46 ± 0.58
LC:V7	QDSKDSTYSLSSTLTLSKADYE	K183	16.0 ± 1.00	16.6 ± 1.01	15.4 ± 0.87	15.7 ± 0.92
LC:V8	KHKVYACE	K188/K190	1.95 ± 0.21	2.00 ± 0.18	1.35 ± 0.27	1.52 ± 0.21
HC:V7	LLGGPSVFLFPPKPKDTLMISRTPE	K250/K252	13.7 ± 0.92	14.2 ± 0.71	14.0 ± 0.80	13.3 ± 0.87
HC:V10	VKFNWYVDGVE	K278	0.81 ± 0.11	0.63 ± 0.13	0.55 ± 0.10	0.78 ± 0.12
HC:V15	KTISKAKGQPREPQVYTLPPSRDE	K338/K342/K344	1.23 ± 0.14	1.15 ± 0.17	1.35 ± 0.16	1.41 ± 0.11
HC:V20	ALHNHYTQKSLSLSPG	K443	0.50 ± 0.14	0.46 ± 0.10	0.42 ± 0.10	0.44 ± 0.12

**Table 4:** Comparison of the expected and experimentally observed masses of the three highly glycated peptides detected in the forced-glycated OR and BS mAb products. Mass errors are shown in parentheses.

Chain: Peptide No	Expected Mass (Da)	Observed Mass (Da) (Mass Error, ppm)			
		OR	BS1	BS2	BS3
LC:V5 (Control)	2001.9989	2001.9983 (−0.2)	2001.9997 (0.4)	2002.0028 (2.0)	2002.0007 (0.9)
LC:V5 (Glycated)	2164.0517	2164.0525 (0.4)	2164.0534 (0.8)	2164.0561 (2.1)	2164.0528 (0.5)
LC:V7 (Control)	2439.1409	2439.1425 (0.6)	2439.1448 (1.6)	2439.1378 (−1.3)	2439.1400 (−0.4)
LC:V7 (Glycated)	2601.1938	2601.1938 (0.0)	2601.1957 (0.7)	2601.1902 (−1.4)	2601.1882 (−2.1)
HC:V7 (Control)	2740.5106	2740.5149 (1.6)	2740.5140 (1.2)	2740.5196 (3.3)	2740.5135 (1.0)
HC:V7 (Glycated)	2902.5634	2902.5640 (0.2)	2902.5616 (−0.6)	2902.5706 (2.5)	2902.5632 (−0.1)

## Data Availability

The data presented in this study are included in the article.

## Supplementary Materials

The following supporting information can be downloaded at: https://www.mdpi.com/article/10.3390/biomedicines14081683/s1, Figure S1: Overlaid raw intact mass spectra of the control OR and BS mAb products; Figure S2: Zoomed-in view of the most intense spectral region in the overlaid raw spectra of the control OR and BS mAb products; Figure S3: Overlaid raw intact mass spectra of the forced-glycated OR and BS mAb products; Figure S4: Zoomed-in view of the most intense spectral region in the overlaid raw spectra of the forced-glycated OR and BS mAb products; Table S1: Comparison of the expected and experimentally observed masses of the six glycated peptides detected in the forced-glycated OR and BS mAb products. Mass errors are shown in parenthesis.

## Abbreviations

The following abbreviations are used in this manuscript:

AGE	Advanced glycation end product
BAC	Boronate affinity chromatography
BS	Biosimilar
CDR	Complementarity-determining region
CHO	Chinese hamster ovary
CID	Collision-induced dissociation
cIEF	Capillary isoelectric focusing
CQA	Critical quality attribute
Da	Dalton
DTT	Dithiothreitol
E	Glutamic acid
EMA	European Medicines Agency
ESI	Electrospray ionization
FA	Formic acid
Fc	Fragment crystallizable
FDA	Food and Drug Administration
HC	Heavy chain
IAM	Iodoacetamide
IEX	Ion-exchange chromatography
IgG	Immunoglobulin G
K	Lysine
kDa	Kilo dalton
LC	Light chain
LC–MS	Liquid chromatography-mass spectrometry
LC–MS/MS	Liquid chromatography-tandem mass spectrometry
mAb	Monoclonal antibody
MaxEnt1	Maximum entropy
MS	Mass spectrometry
MWCO	Molecular weight cut off
NMR	Nuclear magnetic resonance
OR	Originator
QToF	Quadrupole time-of-flight
ppm	Parts-per million
PTM	Post-translational modification
SD	Standard deviation
TFA	Trifluoracetic acid
TIC	Total ion chromatogram
TNF-α	Tumor necrosis factor-alpha
UV	Ultraviolet
XIC	Extracted ion chromatogram
