# Supplementary material for: Mass Spectrometry-Based Comparative Evaluation of Forced Glycation Profiles of Therapeutic Monoclonal Antibodies
Source: Biomedicines. 2026 Jul 27;14(8):1683. doi: 10.3390/biomedicines14081683 (PMC13509498; doi:10.3390/biomedicines14081683)
Supplement: Supplementary file 1 [file biomedicines-14-01683-s001.zip › biomedicines-4386518-supplementary.pdf]

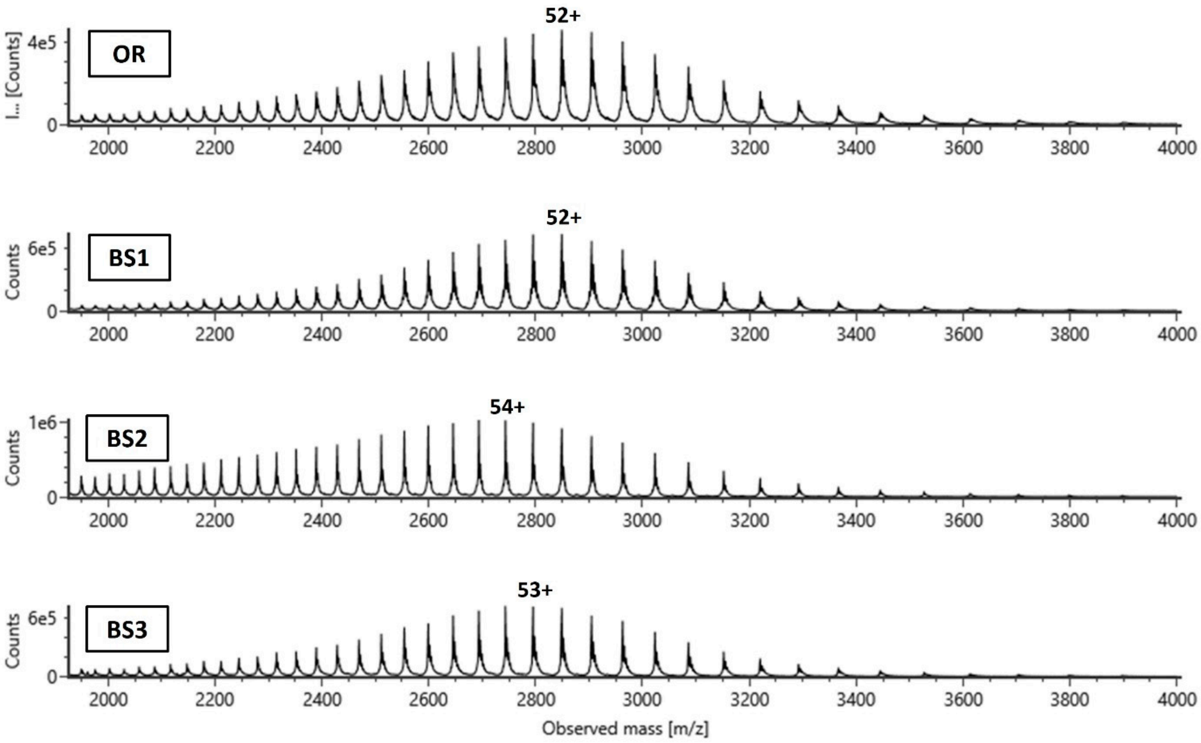

Figure S1. Overlaid raw intact mass spectra of the control OR and BS mAb products

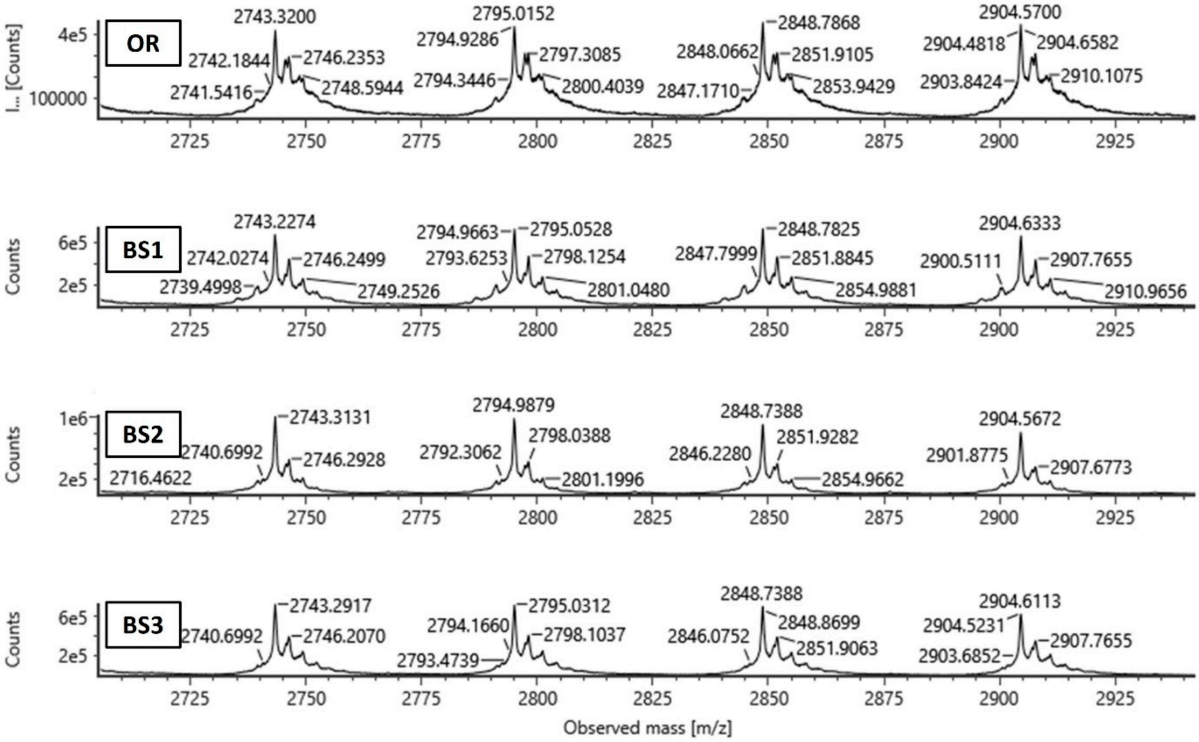

Figure S2. Zoomed-in view of the most intense spectral region in the overlaid raw spectra of the control OR and BS mAb products

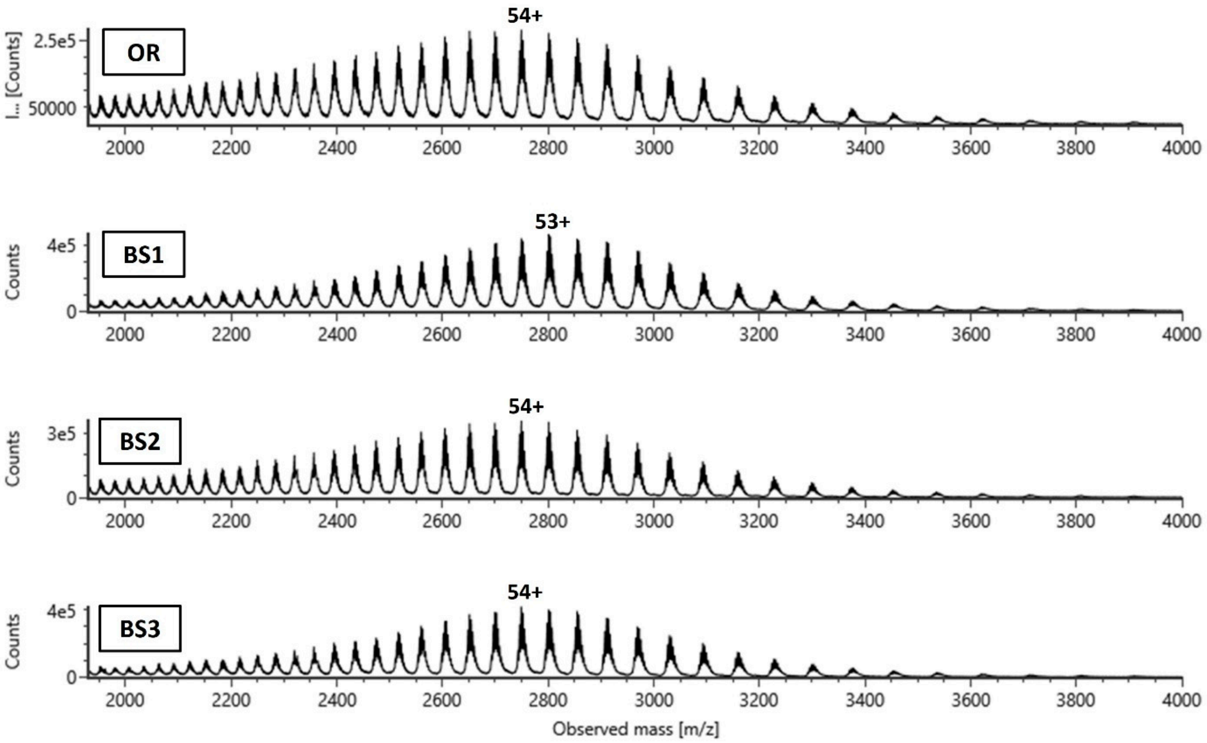

Figure S3. Overlaid raw intact mass spectra of the forced-glycated OR and BS mAb products

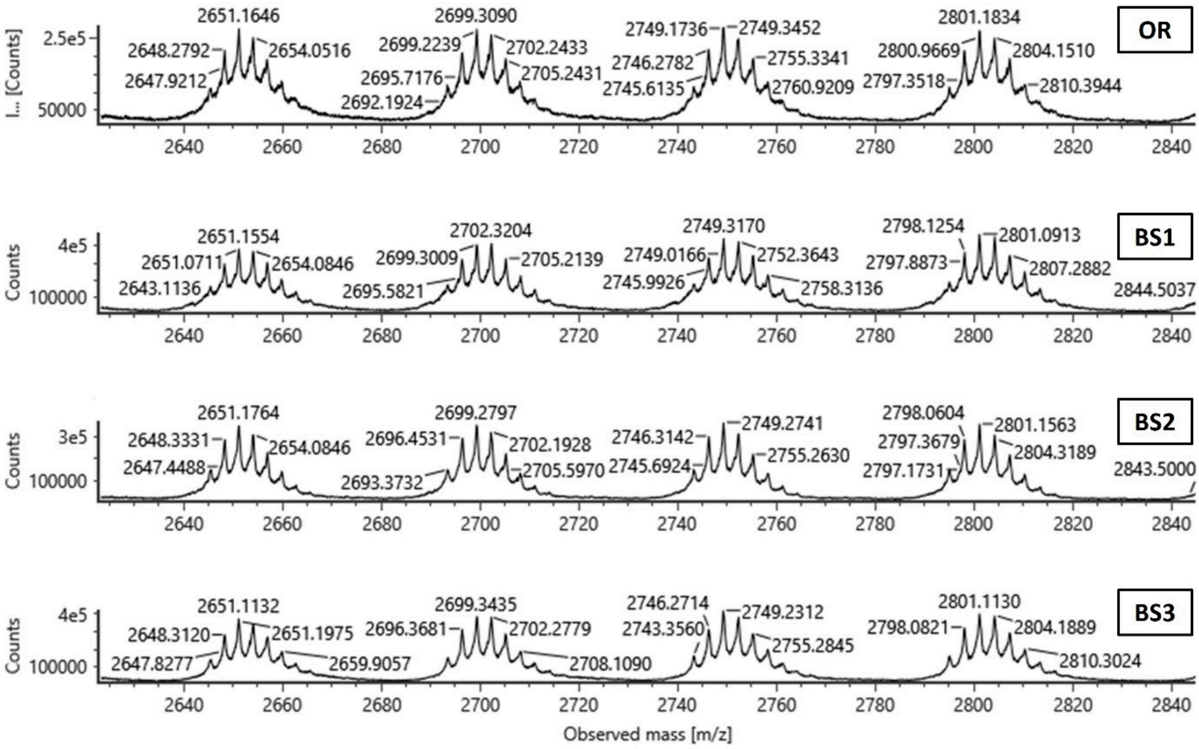

Figure S4. Zoomed-in view of the most intense spectral region in the overlaid raw spectra of the forced-glycated OR and BS mAb products

Table S1. Comparison of the expected and experimentally observed masses of the six glycated peptides detected in the forced-glycated OR and BS mAb products. Mass errors are shown in parenthesis.

Chain: Peptide No	Expected Mass (Da)	Observed Mass (Da) (Mass Error, ppm)			
		OR	BS1	BS2	BS3
LC: V3 (Control)	1974.0695	1974.0706 (0.5)	1974.0712 (0.9)	1974.0715 (1.0)	1974.0737 (2.1)
LC: V3 (Glycated)	2136.1223	2136.1207 (−0.8)	2136.1211 (−0.6)	2136.1240 (0.8)	2136.1220 (−0.1)
LC: V4 (Control)	2296.1754	2296.1716 (−1.7)	2296.1701 (−2.3)	2296.1767 (0.6)	2296.1757 (0.1)
LC: V4 (Glycated)	2458.2283	2458.2194 (−3.6)	2458.2192 (−3.7)	2458.2239 (−1.7)	2458.2314 (1.3)
LC: V8 (Control)	1034.5088	1034.5115 (2.6)	1034.5109 (2.1)	1034.5079 (−0.9)	1034.5110 (2.1)
LC: V8 (Glycated)	1196.5616	1196.5630 (1.2)	1196.5635 (1.6)	1196.5589 (−2.3)	1196.5633 (1.5)
HC: V10 (Control)	1355.6630	1355.6631 (0.1)	1355.6648 (1.3)	1355.6662 (2.4)	1355.6647 (1.2)
HC: V10 (Glycated)	1517.7159	1517.7154 (−0.3)	1517.7158 (−0.1)	1517.7180 (1.4)	1517.7159 (0.0)
HC: V15 (Control)	2725.4632	2725.4686 (2.0)	2725.4674 (1.5)	2725.4699 (2.5)	2725.4650 (0.7)
HC: V15 (Glycated)	2887.5160	2887.5106 (−1.8)	2887.5106 (−1.9)	2887.5114 (−1.6)	2887.5054 (−3.7)
HC: V20 (Control)	1752.9028	1752.9035 (0.4)	1752.9065 (2.1)	1752.9059 (1.8)	1752.9055 (1.6)
HC: V20 (Glycated)	1914.9556	1914.9561 (0.3)	1914.9565 (0.5)	1914.9571 (0.8)	1914.9536 (−1.0)
